# Supplementary material for: Multivalent cross-linking of actin filaments and microtubules through the microtubule-associated protein Tau
Source: Nat Commun. 2017 Dec 7;8:1981. doi: 10.1038/s41467-017-02230-8 (PMC5719408; doi:10.1038/s41467-017-02230-8)
Supplement: Supplementary file 1 — Supplementary Info [file 41467_2017_2230_MOESM1_ESM.pdf]

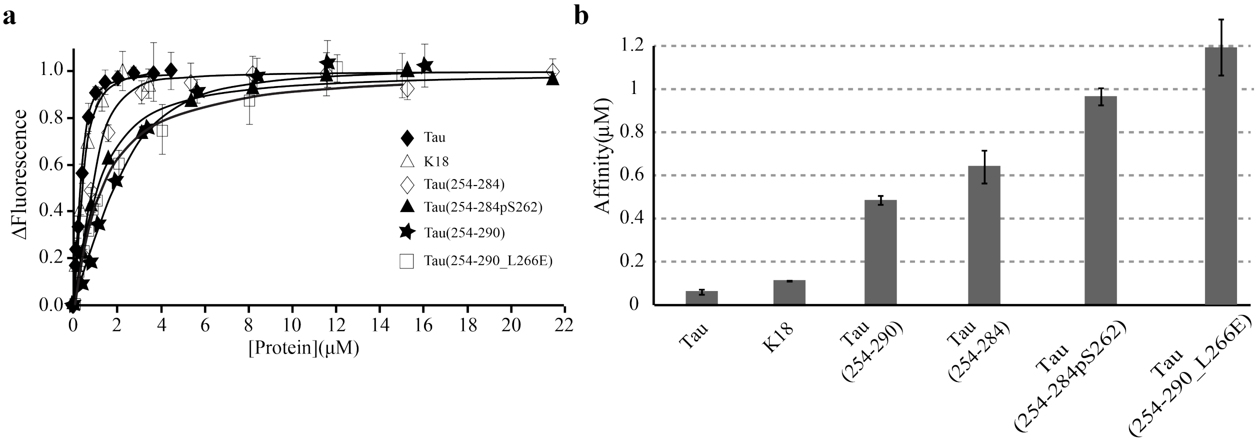

Supplementary Figure 1. Affinity of Tau and Tau fragments for binding to NBD-labeled F-actin. (a) Change of NBD fluorescence upon addition of increasing concentrations of Tau and Tau fragments. Concentrations of F-actin derived from the fit were 0.51 μM (Tau), 0.30 μM (K18), 0.25 μM (Tau(254-284)), 0.25 μM (Tau(254-284pS262)), 2.60 μM (Tau(254-290)) and 0.26 μM (Tau(254-290_L266E)), respectively. **(b)** Bar diagram showing K_d values, which were determined based on the quenching effect of Tau/Tau fragments on the fluorescent properties of NBD-labeled F-actin. Experiments were performed at 25 °C. Error bars represent the standard deviation from three different experiments.

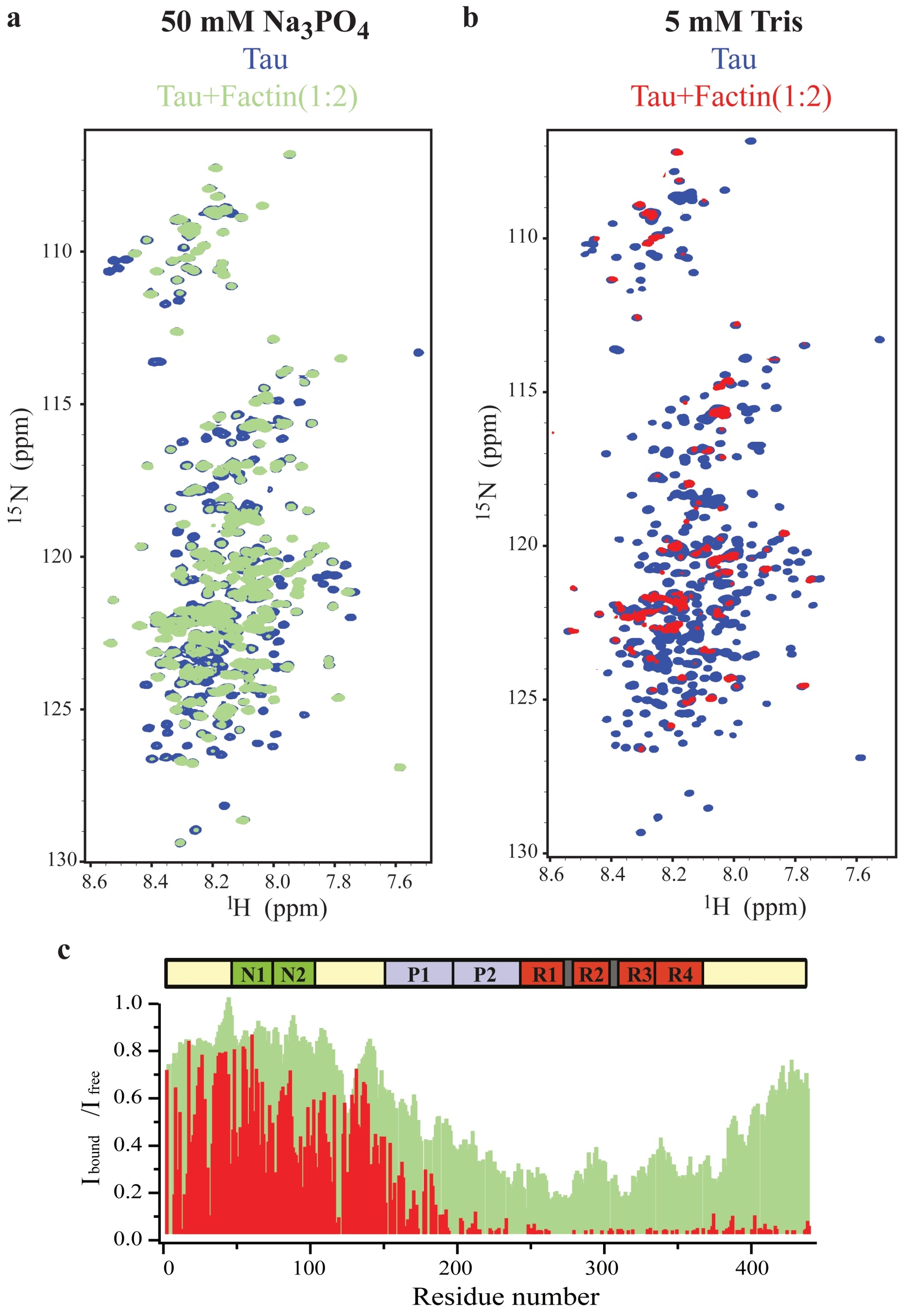

Supplementary Figure 2. ^{1}H-^{15}N HSQC spectra of ^{15}N-labled Tau in the absence (blue) and presence (green/red) of a two-fold excess of F-actin. (a) ^{1}H-^{15}N HSQC spectra in 50 mM sodium phosphate, pH 6.8. **(b)** ^{1}H-^{15}N HSQC spectra in 5mM Tris-HCl, pH 6.8. **(c)** Intensity ratios of cross peaks from the spectra in (a, green) and (b, red) in the presence (I_{bound}) and absence (I_{free}) of F-actin. The domain organization of Tau is shown on top.

a

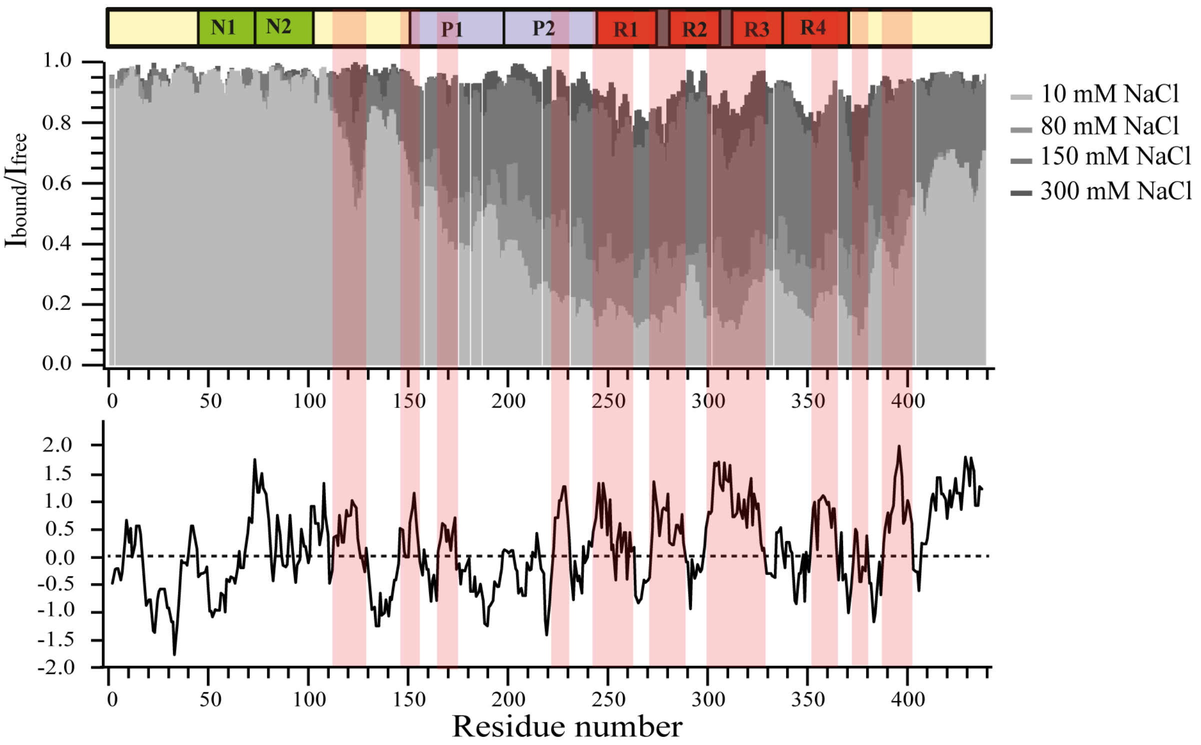

b

R1 [244] Q T A P V P M P D L K - N V K S K I G S T E N L K H Q P G G G - K [274]
R2 [275] V Q I I N K K L D L S - N V Q S K C G S K D N I K H V P G G G - S [305]
R3 [306] V Q I V Y K P V D L S - K V T S K C G S L G N I H H K P G G G - Q [336]
R4 [337] V E V K S E K L D F K D R V Q S K I G S L D N I T H V P G G G N K [369]

Supplementary Figure 3. Ionic strength dependence of the Tau/F-actin interaction. (**a**) Increasing NaCl concentrations decrease F-actin induced signal attenuation in Tau. I_{free} and I_{bound} are signal intensities observed in ^{1}H-^{15}N HSQC spectra of full-length Tau in the absence and presence of a 2-fold excess of F-actin, respectively. The bottom panel represents the residue-specific hydrophobicity profile of Tau. Positive values represent more hydrophobic regions of Tau. (**b**) Sequence alignment of Tau repeats.

a

Residue	Backbone	Sidechain
Cys10	0.00%	0.55%
Cys217	3.68%	0.38%
Cys257	0.00%	0.00%
Cys285	9.07%	0.00%
Cys374	2.22%	44.02%

b

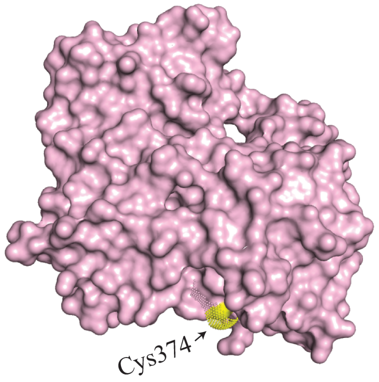

c

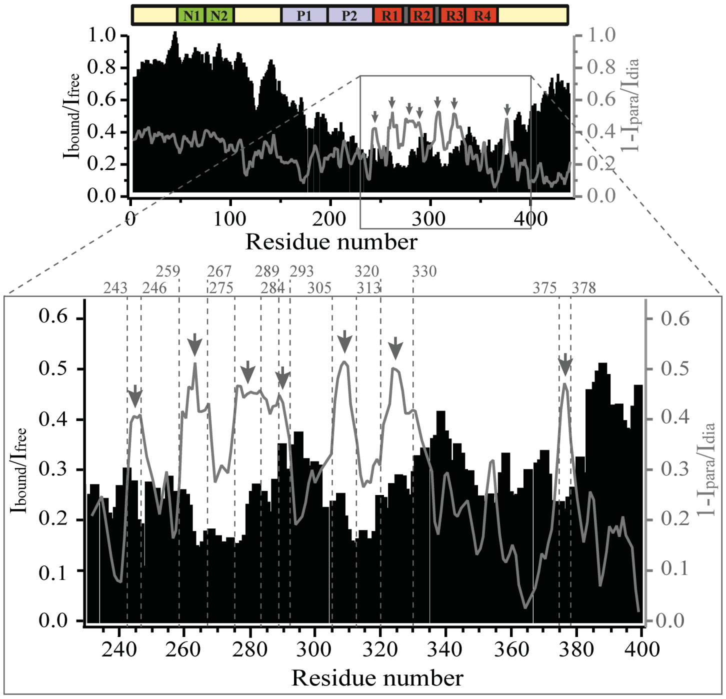

Supplementary Figure 4. Analysis of the solvent accessibility of cysteines in the 3D structure of actin. (a) Table representing the solvent accessibility of the backbone and side chains of actin (PDB id: 3HBT). Values were determined using “Getarea_Remote.pl” (http://curie.utmb.edu/getarea.html). **(b)** Side chain of Cys374 (yellow) is exposed to the solvent according to the crystal structure of actin (PDB id: 3HBT). **(c)** Zoom into the interacting region of Tau with F-actin according to PRE data. I_{para} and I_{dia} are signal intensities observed for individual cross-peaks in two-dimensional ^{1}H-^{15}N HSQCs of Tau in the presence of a two-fold excess of paramagnetic and diagmagnetic F-actin, respectively.

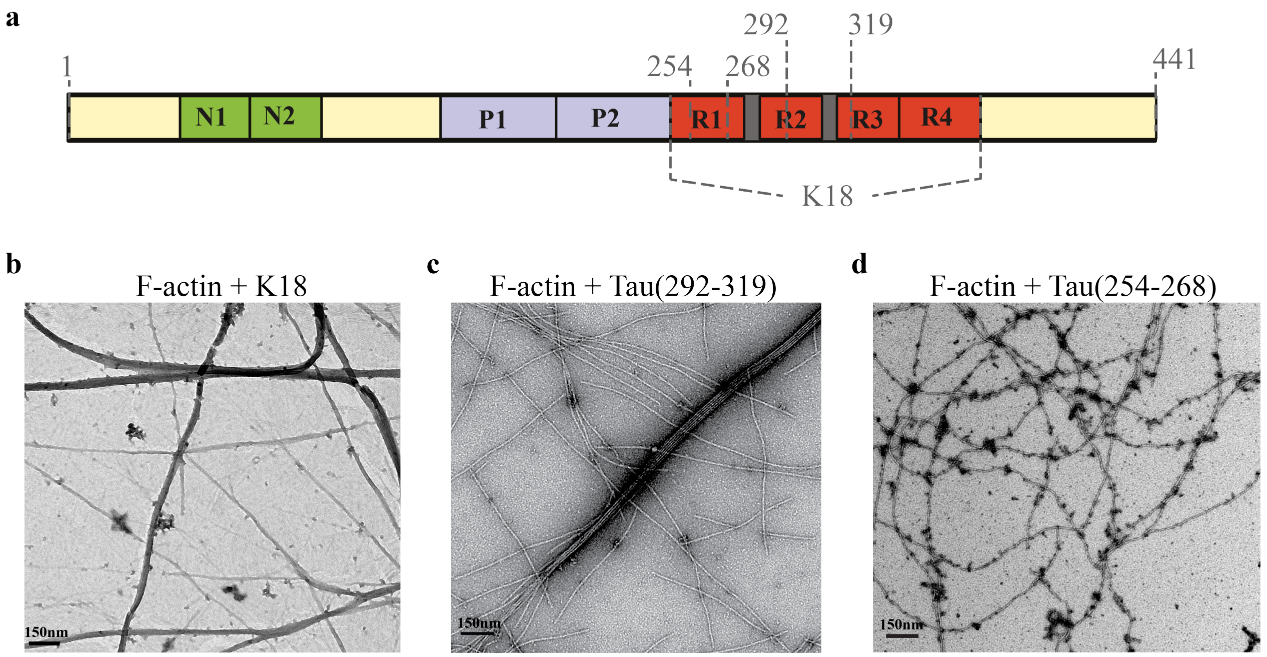

Supplementary Figure 5. Electron microscopy of F-actin in presence of Tau fragments/peptides. (a) Domain organization of the longest isoform of Tau (2N4R), with two insert regions in the projection domain (N1, N2), two proline-rich regions (P1, P2) and the repeat region (R1-R4). Dashed lines delimit the peptides Tau(254-268) and Tau(292-319), as well as the K18 construct, which comprises all four repeats of the largest Tau isoform (residues Gln^{244}-Glu^{372} plus initial Met^{243}). (**b**) 10-fold excess of K18 promotes F-actin bundling. A similar effect was observed with Tau(292-319) (**c**). (**d**) The peptide Tau(254-268), which contains only a single F-actin interaction site, does not promote bundle formation even at 30-fold excess. Bars indicate 150 nm.

a

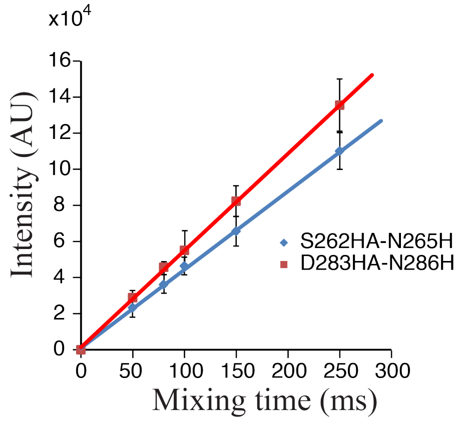

b

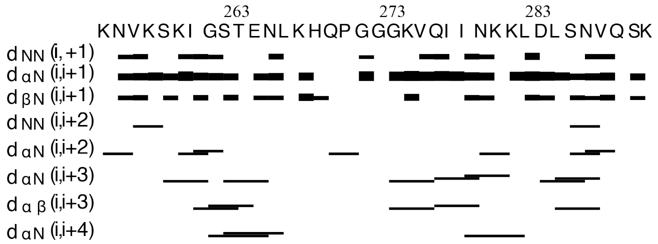

c

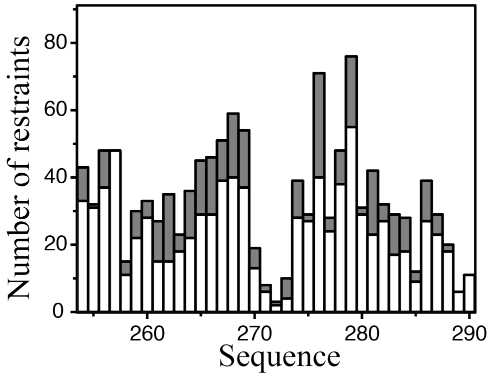

d

Statistics of the calculation for Tau (254-290)

Number of structures: 20

Number of restraints: 182

Intra Residue NOE (|i-j|=0)=0 Medium range NOE (1< |i-j| ≤4)=182

Sequential NOE (|i-j|=1)=0 Long range NOE (|i-j| ≥5)=0

NOE violations > 0.3 Å/structure = 0

RMSD	All residues	residues (256-267)
All backbone atoms	1.9 Å	1.4 Å
All heavy atoms	2.5 Å	2.3 Å

Ramachandran Plot summary	
Most favoured regions:	91.2%
Additionally allowed regions:	6.4%
Generously allowed regions:	0.0%
Disallowed regions:	1.4%

Supplementary Figure 6. Experimental restraints and structural statistics for the lowest-energy conformations of Tau(254-290) bound to F-actin. (**a**) Dependence of NOE intensity on NOE mixing time for the cross-peaks S262HA-N265H (blue) and D283HA-N286H (red). Error bars are estimated from the signal-to-noise ratio in the 2D NOESY spectra. (**b**) Plot of NOE contacts, which are characteristic for regular secondary structure. (**c**) Distribution of distance constraints as a function of residue number. Distance constraints were classified as intra-residual and sequential (|i-j| ≤ 1; white), and medium-range (1 < |i-j| ≤ 4; gray). (**d**) Structural statistics for the 20 lowest-energy conformations of Tau(254-290) bound to F-actin.

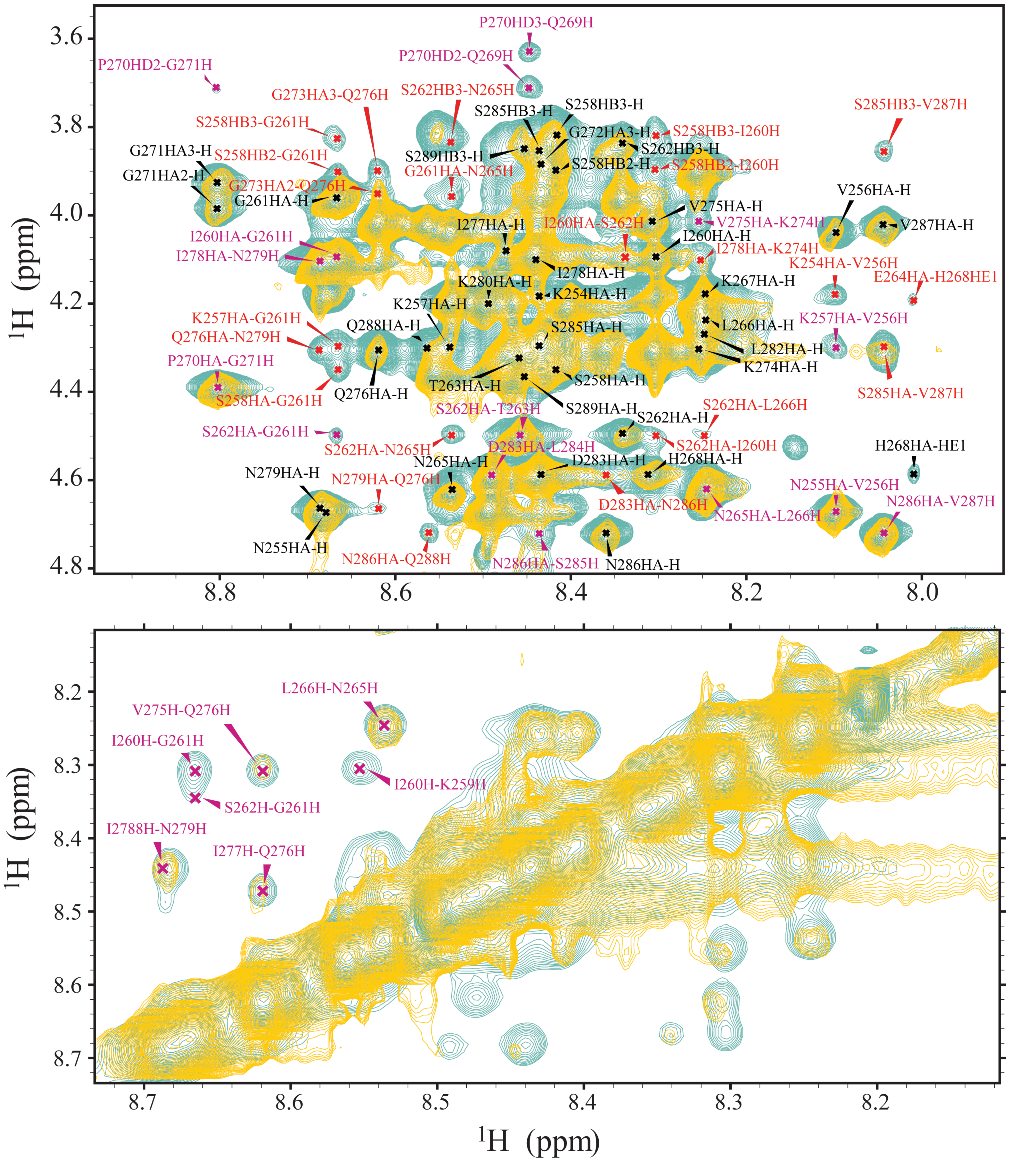

Supplementary Figure 7. Hα-HN (top panel) and HN-HN (bottom panel) regions of the NOE spectrum of Tau(254-290) in the absence (yellow) and presence (green) of F-actin. Cross-peaks corresponding to sequential and medium-range NOEs are highlighted in magenta and red, respectively, while intraresidual peaks are labeled in black.

a

Parameter	Cluster1	Cluster2	Cluster3	Cluster4
Score	1.6±1.1	58.9±9.2	39.6±20.5	82.0±25.7
Cluster size	161	15	10	6
RMSD	1.0±0.8	7.2±0.1	1.8±0.1	8.2±0.1
V.W. Energy	-54.1±8.2	-53.5±9.5	-43.0±10.4	-33.4±2.3
Elect. Energy	-291.9±105.8	-139.2±11.3	-275.4±35.5	-279.7±53.15
Desolvation Energy	110.5±17.1	129.9±7.1	120.6±24.4	156.6±18.1
Restraints Viol. Energy	38.8±8.07	102.5±38.29	170.6±26.04	147.2±3.68

b

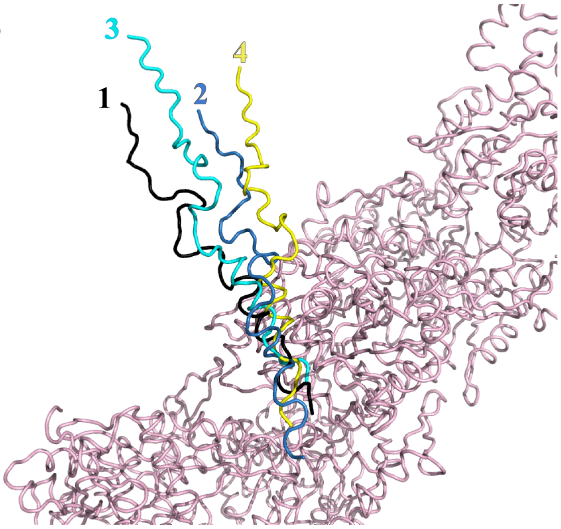

Supplementary Figure 8. Statistics and cluster overlap obtained from docking. (a) Haddock docking statistics for the four identified clusters. **(b)** Ribbon representation of the four different clusters obtained from docking Tau(254-290) to the hydrophobic pocket of actin (magenta). The different clusters are labeled from 1-4 for clarity.

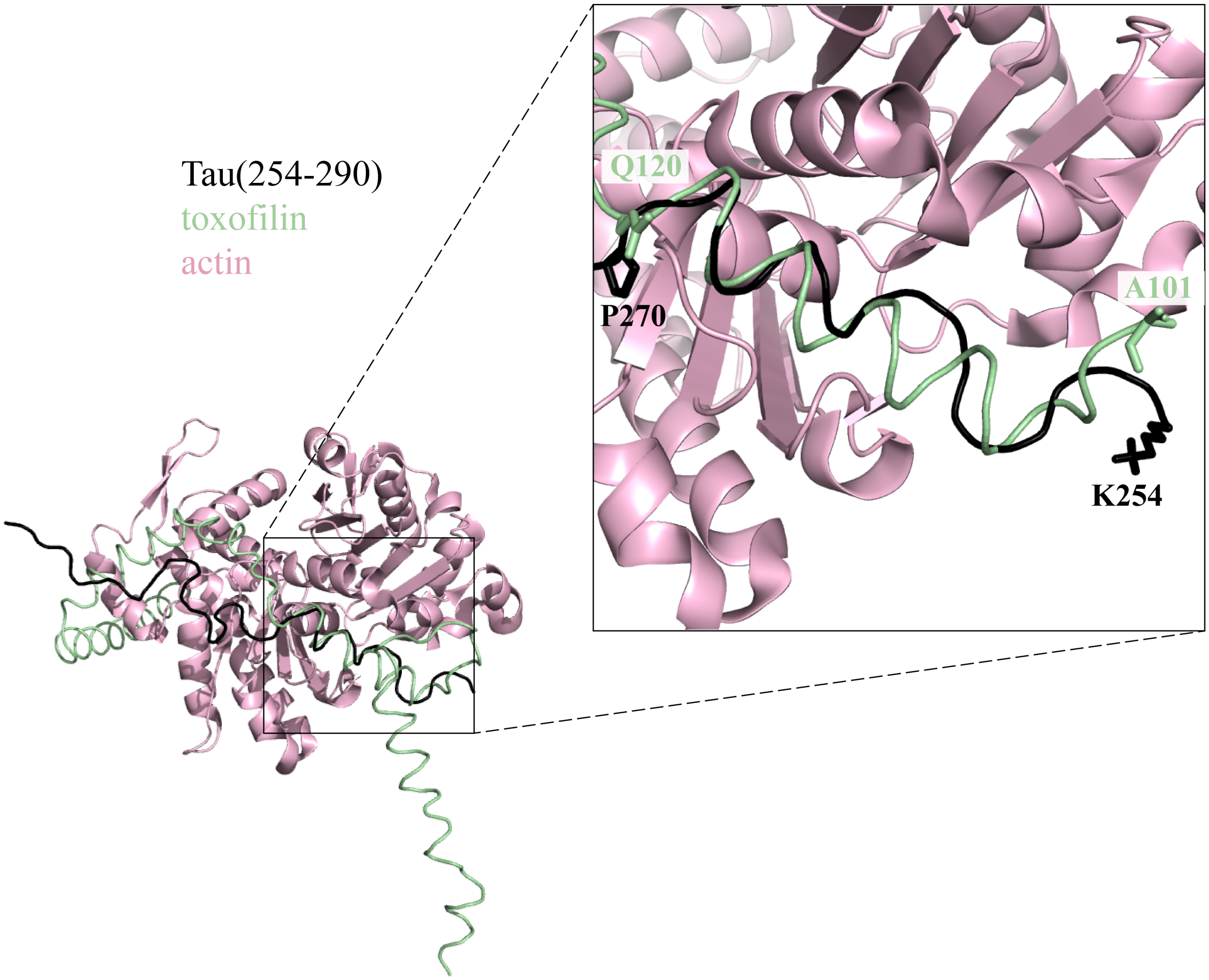

Supplementary Figure 9. Superposition of the crystal structure of toxofilin (green) from *Toxoplasma gondii* in complex with F-actin (pink; PDB id: 2Q97) and the docked peptide Tau(254-290) in black. The inset highlights the superposition of the toxofilin helix with the helical region of Tau(254-290).

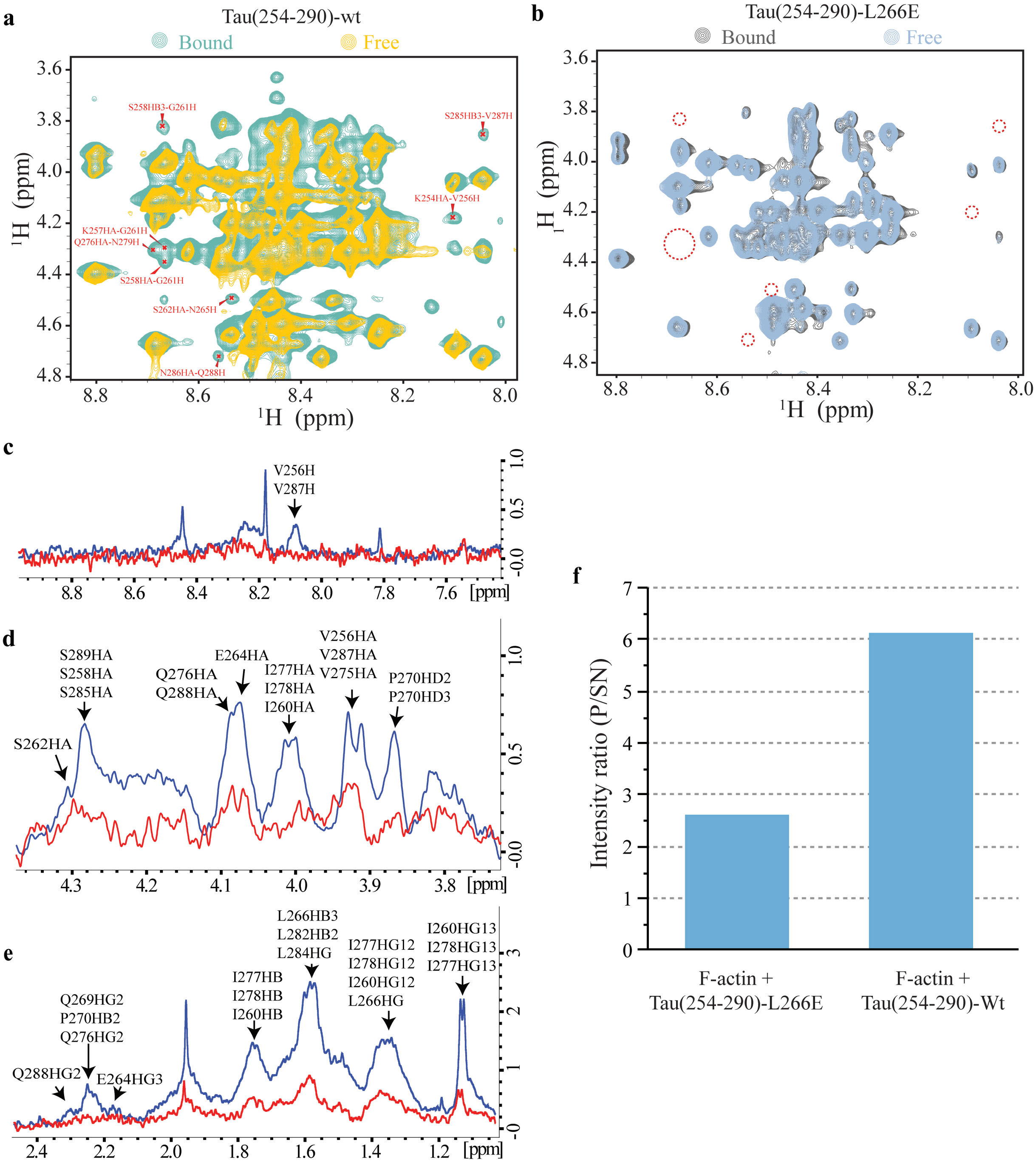

Supplementary Figure 10. NOE and STD analysis of the interaction of wild-type Tau(254-290) and Tau(254-290)-L266E with F-actin. (**a,b**) Selected regions of ^{1}H-^{1}H NOE spectra of Tau(254-290) (a) and Tau(254-290)-L266E (b) in the absence (yellow/blue) and presence (green/grey) of a two-fold molar excess of F-actin. (**c-e**) STD spectra from the amide **(c)**, aliphatic **(d)** and methyl **(e)** regions of wild-type Tau(254-290) (blue) and Tau(254-290)-L266E (red) in the presence of a two-fold molar excess of F-actin. Selected resonance assignments are highlighted. (**f**) Bar diagram representing the bundling properties of Tau(254-290)-L266E and wild-type Tau(254-290) from a co-sedimentation assay. Bars represent the ratio between the bundle-containing pellet (P) fraction and the supernatant (SN), which contains filaments, as derived from the intensities of the two bands observed in a 4-20% gradient SDS gel.

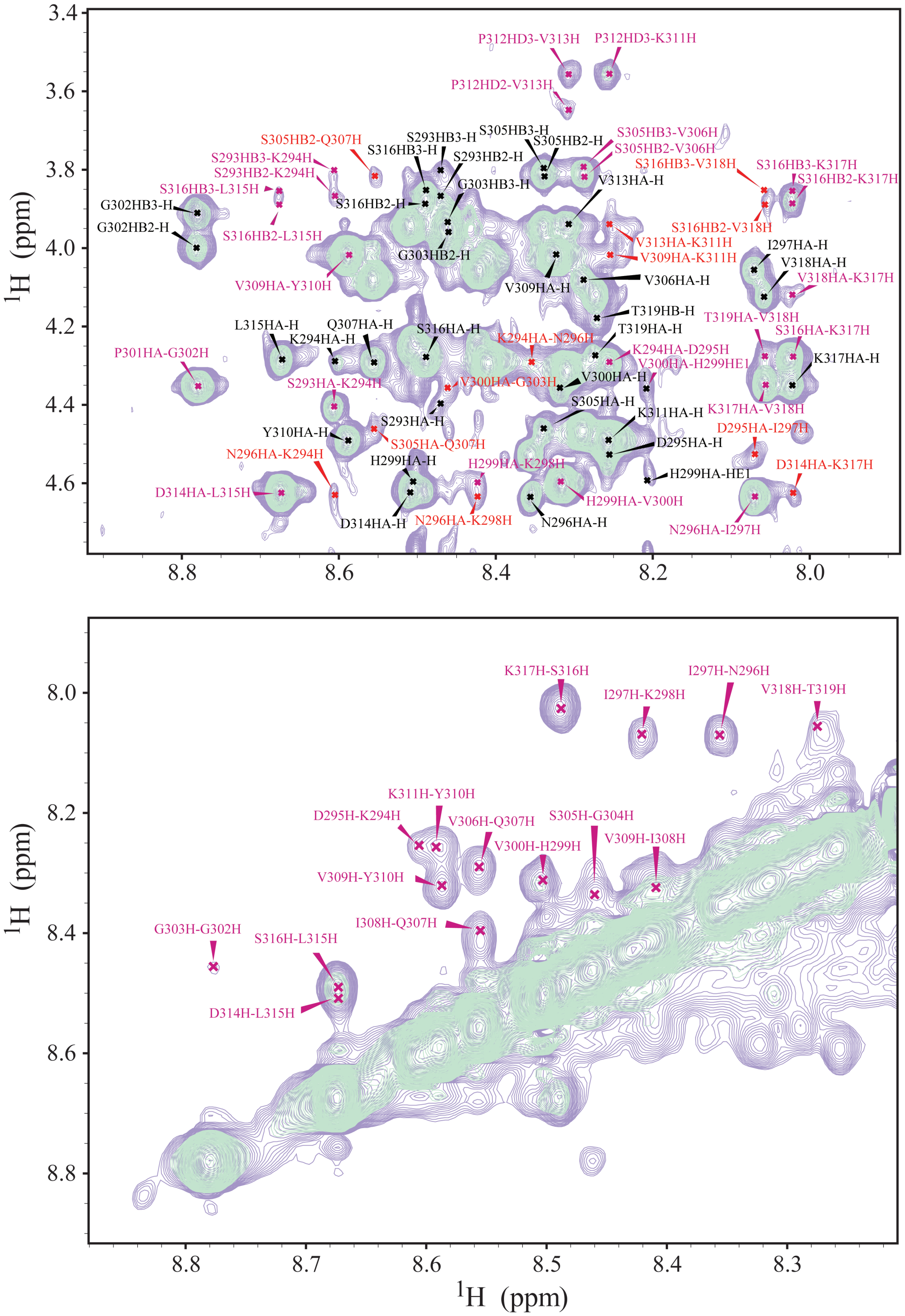

Supplementary Figure 11. Hα-HN (top panel) and HN-HN (bottom panel) regions of the NOE spectrum of Tau(292-319) in the absence (green) and presence (purple) of a two-fold molar excess of F-actin. Cross-peak assignments of Tau(292-319) are indicated.

a

b

c

d

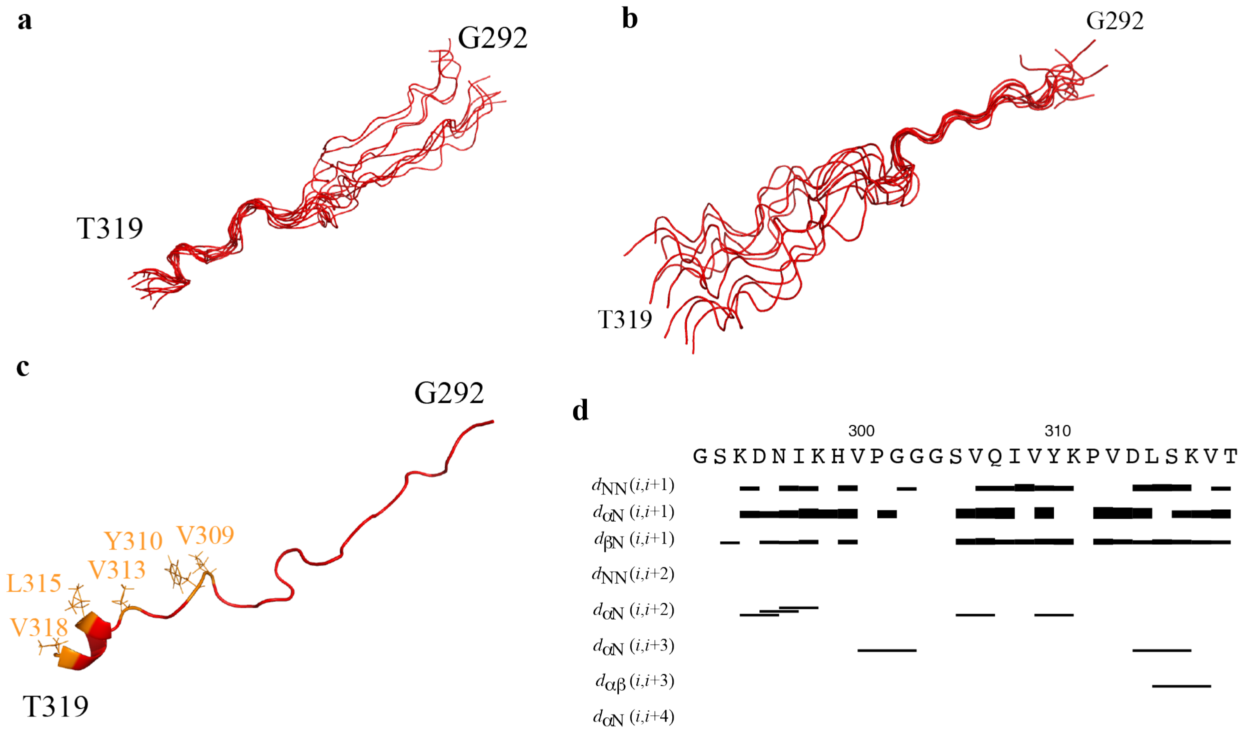

e

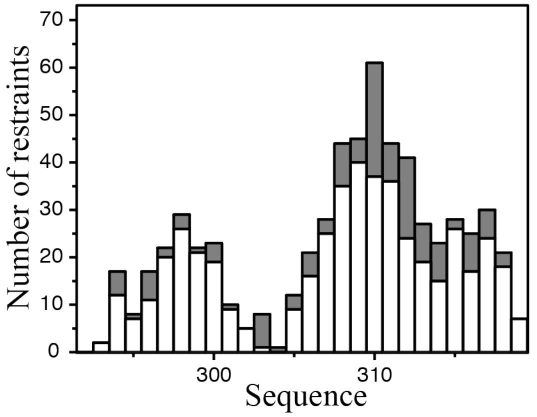

f

Statistics of the calculation for Tau (292-319)

Number of structures: 20
Number of restraints: 70
Intra Residue NOE (|i-j|=0)=0 Medium range NOE (1< |i-j| ≤4)=70
Sequential NOE (|i-j|=1)=0 Long range NOE (|i-j| ≥5)=0
NOE violations > 0.3 Å/structure = 0

RMSD	residues (293-300)	residues (313-319)
All backbone atoms	0.53 Å	0.83 Å
All heavy atoms	1.35 Å	1.49 Å

Ramachandran Plot summary

Most favoured regions:	71.9%
Additionally allowed regions:	14.3%
Generously allowed regions:	8.1%
Disallowed regions:	5.7%

Supplementary Figure 12. Tau(292-319) bound to F-actin. (**a**,**b**) 10 lowest-energy conformers of F-actin-bound Tau(292-319) aligned to each other with respect to the C- and N-terminus, respectively. (**c**) Schematic representation of the N-terminal helical region. Side-chains of hydrophobic residues are shown. (**d**) Plot of NOE contacts, which are characteristic for regular secondary structure. (**e**) Distribution of distance constraints as a function of residue number. Distance constraints were classified as intra-residual and sequential (|i-j| ≤ 1; white), and medium-range (1 < |i-j| ≤ 4; gray). (**f**) Structural statistics for the 20 lowest-energy conformations of Tau(292-319) bound to F-actin.

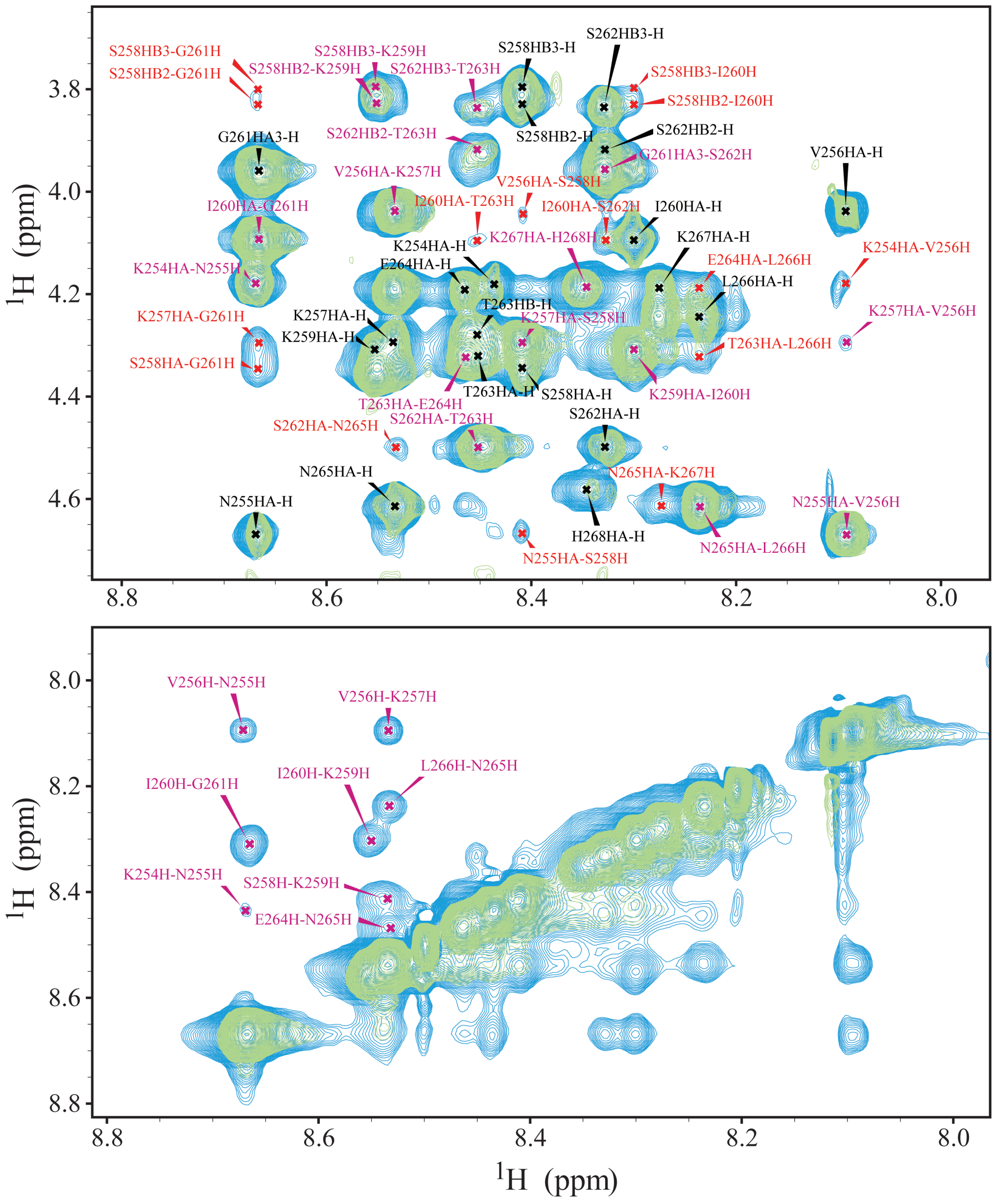

Supplementary Figure 13. Hα-HN (top panel) and HN-HN (bottom panel) regions of the NOE spectrum of the peptide Tau(254-268) in absence (green) and presence (blue) of F-actin. Cross-peaks corresponding to sequential and medium-range interaction NOEs are highlighted in magenta and red, respectively, while intraresidual peaks are colored in black

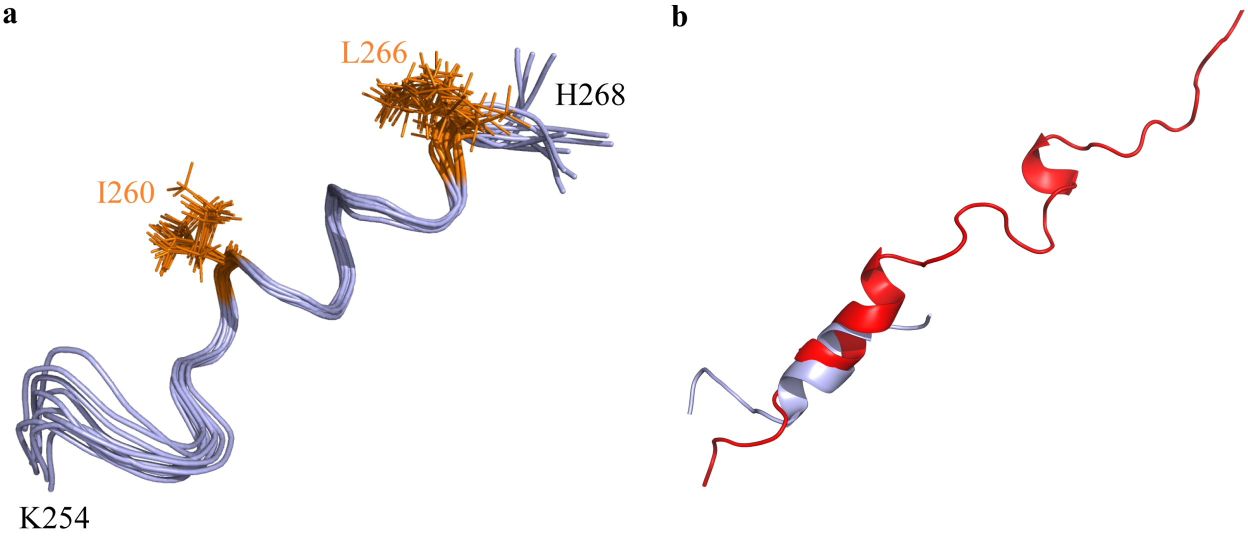

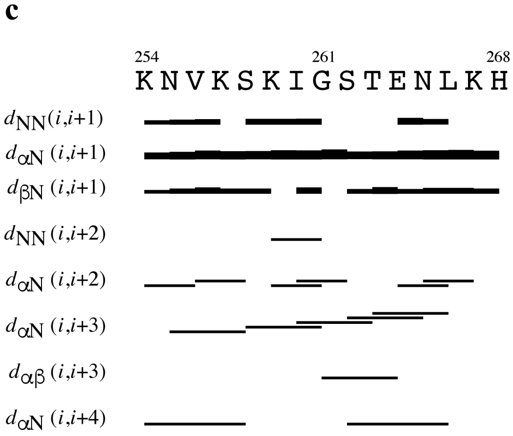

d

Statistics of the calculation for Tau (254-268)	
Number of structures: 20	
Number of restraints: 38	
Intra Residue NOE (|i-j|=0)=0	Medium range NOE (1< |i-j| ≤4)=38
Sequential NOE (|i-j|=1)=0	Long range NOE (|i-j| ≥5)=0
NOE violations > 0.3 Å/structure = 0	
RMSD	All residues
All backbone atoms	0.99 Å
All heavy atoms	1.63 Å
Ramachandran Plot summary	
Most favoured regions:	54.2%
Additionally allowed regions:	41.7%
Generously allowed regions:	4.2%
Disallowed regions:	0.0%

Supplementary Figure 14. Structure of Tau(254-268) in complex with F-actin. (**a**) 10 lowest-energy conformations of Tau(254-268), when the peptide is bound to F-actin. The structures were calculated on the basis of 38 medium-range distance restraints, which were derived from NOE spectra in the presence of F-actin (molar ratio of Tau(254-268):F-actin of 30:1). (**b**) Superposition of the helix of F-actin bound Tau(254-268) with the same region in F-actin bound Tau(254-290). **(c)** Plot of NOE contacts, which are characteristic for regular secondary structure. **(d)** Structural statistics for the 20 lowest-energy conformations of Tau(254-368) bound to F-actin.

Supplementary Table 1. Resonance assignment of Tau(254-290)

Group	Atom	Nuc.	Shift
K254	H	1H	8.436
K254	HA	1H	4.181
K254	HB2	1H	1.746
K254	HB3	1H	1.686
K254	HD	1H	1.627
K254	HE3	1H	2.945
K254	HG2	1H	1.423
K254	HG3	1H	1.361
N255	H	1H	8.678
N255	HA	1H	4.673
N255	HB2	1H	2.800
N255	HB3	1H	2.700
N255	HD21	1H	7.713
N255	HD22	1H	6.991
V256	H	1H	8.098
V256	HA	1H	4.035
V256	HB	1H	2.040
V256	HG1	1H	0.879
V256	HG2	1H	0.821
K257	H	1H	8.537
K257	HA	1H	4.297
K257	HB2	1H	1.803
K257	HB3	1H	1.719
K257	HD	1H	1.637
K257	HE3	1H	2.944
K257	HG2	1H	1.426
K257	HG3	1H	1.375
S258	H	1H	8.420
S258	HA	1H	4.350
S258	HB2	1H	3.899
S258	HB3	1H	3.812
K259	H	1H	8.553
K259	HA	1H	4.306
K259	HB2	1H	1.812
K259	HB3	1H	1.710
K259	HD	1H	1.634
K259	HE3	1H	2.950
K259	HG2	1H	1.419
K259	HG3	1H	1.370
I260	H	1H	8.304
I260	HA	1H	4.093
I260	HB	1H	1.813
I260	HD1	1H	0.819
I260	HG12	1H	1.460
I260	HG13	1H	1.166
I260	HG2	1H	0.887
G261	H	1H	8.666
G261	HA	1H	3.957
S262	H	1H	8.341
S262	HA	1H	4.497
S262	HB2	1H	3.922
S262	HB3	1H	3.835
T263	H	1H	8.459
T263	HA	1H	4.322
T263	HB	1H	4.271
T263	HG2	1H	1.175
E264	H	1H	8.463
E264	HA	1H	4.191
E264	HB2	1H	1.982
E264	HB3	1H	1.891
E264	HG2	1H	2.230
E264	HG3	1H	2.201
N265	H	1H	8.536
N265	HA	1H	4.620
N265	HB2	1H	2.799
N265	HB3	1H	2.693
N265	HD21	1H	7.689
N265	HD22	1H	6.976
L266	H	1H	8.247
L266	HA	1H	4.246
L266	HB2	1H	1.676
L266	HB3	1H	1.582
L266	HD1	1H	0.884
L266	HD2	1H	0.812
L266	HG	1H	1.493
K267	H	1H	8.247
K267	HA	1H	4.178
K267	HB2	1H	1.687
K267	HB3	1H	1.613
K267	HD	1H	1.555
K267	HD3	1H	1.613
K267	HE3	1H	2.919
K267	HG2	1H	1.345
K267	HG3	1H	1.275
H268	H	1H	8.312
H268	HA	1H	4.583
H268	HB2	1H	3.094
H268	HB3	1H	3.025
H268	HD2	1H	7.014
H268	HE1	1H	8.009
Q269	H	1H	8.448
Q269	HA	1H	4.577
Q269	HB2	1H	2.027
Q269	HB3	1H	1.841
Q269	HE21	1H	7.599
Q269	HE22	1H	6.971
Q269	HG	1H	2.306
Q269	HG2	1H	2.304

Group	Atom	Nuc.	Shift
P270	HA	1H	4.389
P270	HB2	1H	2.287
P270	HB3	1H	2.034
P270	HD2	1H	3.711
P270	HD3	1H	3.631
P270	HG2	1H	1.979
P270	HG3	1H	1.922
G271	H	1H	8.802
G271	HA2	1H	3.985
G271	HA3	1H	3.926
G272	H	1H	8.435
G272	HA2	1H	3.943
G272	HA3	1H	3.884
G273	H	1H	8.433
G273	HA2	1H	3.950
G273	HA3	1H	3.894
K274	H	1H	8.253
K274	HA	1H	4.302
K274	HB2	1H	1.774
K274	HB3	1H	1.694
K274	HD	1H	1.627
K274	HE3	1H	2.940
K274	HG2	1H	1.415
K274	HG3	1H	1.343
V275	H	1H	8.307
V275	HA	1H	4.011
V275	HB	1H	1.969
V275	HG1	1H	0.89
V275	HG2	1H	0.808
Q276	H	1H	8.620
Q276	HA	1H	4.304
Q276	HB2	1H	1.978
Q276	HB3	1H	1.895
Q276	HE21	1H	7.613
Q276	HE22	1H	6.968
Q276	HG	1H	2.286
I277	H	1H	8.474
I277	HA	1H	4.082
I277	HB	1H	1.780
I277	HD1	1H	0.812
I277	HG12	1H	1.442
I277	HG13	1H	1.141
I277	HG2	1H	0.885
I278	H	1H	8.440
I278	HA	1H	4.101
I278	HB	1H	1.784
I278	HD1	1H	0.818
I278	HG12	1H	1.424
I278	HG13	1H	1.132
I278	HG2	1H	0.887
N279	H	1H	8.686

Group	Atom	Nuc.	Shift
N279	HA	1H	4.663
N279	HB2	1H	2.791
N279	HD21	1H	7.713
N279	HD22	1H	6.973
N279	HB3	1H	2.679
K280	H	1H	8.493
K280	HA	1H	4.200
K280	HB2	1H	1.786
K280	HB3	1H	1.691
K280	HD3	1H	1.692
K280	HE3	1H	2.946
K280	HG2	1H	1.414
K280	HG3	1H	1.365
K281	H	1H	8.405
K281	HA	1H	4.200
K281	HB2	1H	1.787
K281	HB3	1H	1.719
K281	HD	1H	1.646
K281	HE3	1H	2.949
K281	HG2	1H	1.416
K281	HG3	1H	1.357
L282	H	1H	8.248
L282	HA	1H	4.268
L282	HB2	1H	1.731
L282	HB3	1H	1.568
L282	HD1	1H	0.89
L282	HD2	1H	0.819
L282	HG	1H	1.505
D283	H	1H	8.435
D283	HA	1H	4.588
D283	HB2	1H	2.751
D283	HB3	1H	2.558
L284	H	1H	8.490
L284	HA	1H	4.322
L284	HB2	1H	1.810
L284	HB3	1H	1.711
L284	HD1	1H	0.892
L284	HD2	1H	0.813
L284	HG	1H	1.610
S285	H	1H	8.435
S285	HA	1H	4.297
S285	HB2	1H	3.949
S285	HB3	1H	3.856
N286	H	1H	8.359
N286	HA	1H	4.719
N286	HB2	1H	2.819
N286	HB3	1H	2.710
N286	HD21	1H	7.707
N286	HD22	1H	6.934
V287	H	1H	8.043
V287	HA	1H	4.020

Group	Atom	Nuc.	Shift
V287	HB	1H	2.061
V287	HG1	1H	0.906
V287	HG2	1H	0.818
Q288	H	1H	8.563
Q288	HA	1H	4.298
Q288	HB2	1H	2.076
Q288	HB3	1H	1.965
Q288	HE21	1H	7.603
Q288	HE22	1H	6.929
Q288	HG	1H	2.356
S289	H	1H	8.453

Group	Atom	Nuc.	Shift
S289	HA	1H	4.366
S289	HB2	1H	3.950
S289	HB3	1H	3.850
K290	H	1H	8.476
K290	HA	1H	4.259
K290	HB2	1H	1.847
K290	HB3	1H	1.742
K290	HE3	1H	2.945
K290	HG2	1H	1.443
K290	HG3	1H	1.384

Supplementary Table 2. Resonance assignment of Tau(292-319)

Group	Atom	Nuc.	Shift
G292	H	1H	8.503
G292	HB2	1H	3.953
G292	HB3	1H	3.921
S293	H	1H	8.470
S293	HA	1H	4.395
S293	HB2	1H	3.868
S293	HB3	1H	3.802
K294	H	1H	8.606
K294	HA	1H	4.289
K294	HB2	1H	1.840
K294	HB3	1H	1.729
K294	HD2	1H	1.633
K294	HE2	1H	2.944
K294	HG2	1H	1.414
K294	HG3	1H	1.364
D295	H	1H	8.255
D295	HA	1H	4.527
D295	HB2	1H	2.657
D295	HB3	1H	2.548
N296	H	1H	8.356
N296	HA	1H	4.634
N296	HB2	1H	2.781
N296	HB3	1H	2.694
N296	HD21	1H	7.665
N296	HD22	1H	6.463
I297	H	1H	8.070
I297	HA	1H	4.053
I297	HB	1H	1.805
I297	HD1	1H	0.768
I297	HG12	1H	1.388
I297	HG13	1H	1.121
I297	HG2	1H	0.807
K298	H	1H	8.422
K298	HA	1H	4.237
K298	HB2	1H	1.710
K298	HB3	1H	1.669
K298	HD2	1H	1.612
K298	HE2	1H	2.927
K298	HG2	1H	1.374
K298	HG3	1H	1.292
H299	H	1H	8.504
H299	HA	1H	4.596
H299	HB2	1H	3.052
H299	HB3	1H	3.021
H299	HD2	1H	7.014
H299	HE1	1H	8.207
V300	H	1H	8.317
V300	HA	1H	4.357
V300	HB	1H	1.993
V300	HG1	1H	0.889
Group	Atom	Nuc.	Shift

Group	Atom	Nuc.	Shift
V300	HG2	1H	0.851
P301	HA	1H	4.352
P301	HB2	1H	2.300
P301	HB3	1H	2.045
P301	HD1	1H	3.639
P301	HD2	1H	3.804
P301	HD3	1H	3.642
P301	HG2	1H	1.951
P301	HG3	1H	1.902
G302	H	1H	8.779
G302	HB2	1H	4.002
G302	HB3	1H	3.910
G303	H	1H	8.461
G303	HB2	1H	3.959
G303	HB3	1H	3.933
G304	H	1H	8.432
G304	HB2	1H	3.955
G304	HB3	1H	3.933
S305	H	1H	8.338
S305	HA	1H	4.462
S305	HB2	1H	3.818
S305	HB3	1H	3.795
V306	H	1H	8.288
V306	HA	1H	4.080
V306	HB	1H	2.005
V306	HG1	1H	0.886
V306	HG2	1H	0.87
Q307	H	1H	8.555
Q307	HA	1H	4.292
Q307	HB2	1H	1.969
Q307	HB3	1H	1.889
Q307	HE21	1H	7.591
Q307	HE22	1H	6.937
Q307	HG2	1H	2.299
Q307	HG3	1H	2.263
I308	H	1H	8.409
I308	HA	1H	4.012
I308	HB	1H	1.692
I308	HD1	1H	0.648
I308	HG12	1H	1.411
I308	HG13	1H	1.089
I308	HG2	1H	0.783
V309	H	1H	8.323
V309	HA	1H	4.016
V309	HB	1H	1.908
V309	HG1	1H	0.858
V309	HG2	1H	0.821
Y310	H	1H	8.588
Y310	HA	1H	4.491
Y310	HB2	1H	2.895
Group	Atom	Nuc.	Shift

Y310	HB3	1H	2.865
Y310	HD1	1H	7.060
Y310	HE1	1H	6.740
K311	H	1H	8.257
K311	HA	1H	4.491
K311	HB2	1H	1.693
K311	HB3	1H	1.616
K311	HD2	1H	1.579
K311	HE2	1H	2.917
K311	HG2	1H	1.358
K311	HG3	1H	1.307
P312	HA	1H	4.309
P312	HB2	1H	2.258
P312	HB3	1H	1.969
P312	HD2	1H	3.566
P312	HD3	1H	3.543
P312	HG2	1H	1.948
P312	HG3	1H	1.849
V313	H	1H	8.307
V313	HA	1H	3.943
V313	HB	1H	1.964
V313	HG1	1H	0.922
V313	HG2	1H	0.87
D314	H	1H	8.508
D314	HA	1H	4.624
D314	HB2	1H	2.759
D314	HB3	1H	2.552

L315	H	1H	8.673
L315	HA	1H	4.286
L315	HB2	1H	1.658
L315	HB3	1H	1.613
L315	HD1	1H	0.898
L315	HD2	1H	0.81
S316	H	1H	8.490
S316	HA	1H	4.277
S316	HB2	1H	3.889
S316	HB3	1H	3.853
K317	H	1H	8.022
K317	HA	1H	4.348
K317	HB2	1H	1.858
K317	HB3	1H	1.701
K317	HD2	1H	1.619
K317	HE2	1H	2.939
K317	HG2	1H	1.405
K317	HG3	1H	1.318
V318	H	1H	8.057
V318	HA	1H	4.120
V318	HB	1H	2.085
V318	HG1	1H	0.921
V318	HG2	1H	0.904
T319	H	1H	8.272
T319	HA	1H	4.273
T319	HB	1H	4.178
T319	HG2	1H	1.168

Supplementary Table 3. Resonance assignment of Tau(254-268)

Group	Atom	Nuc.	Shift
K254	H	1H	8.435
K254	HA	1H	4.174
K254	HB2	1H	1.747
K254	HB3	1H	1.686
K254	HD2	1H	1.641
K254	HE2	1H	2.944
K254	HG2	1H	1.410
K254	HG3	1H	1.368
N255	H	1H	8.669
N255	HA	1H	4.667
N255	HB2	1H	2.800
N255	HB3	1H	2.703
N255	HD21	1H	7.706
N255	HD22	1H	6.986
V256	H	1H	8.093
V256	HA	1H	4.037
V256	HB	1H	2.040
V256	HG1	1H	0.877
V256	HG2	1H	0.808
K257	H	1H	8.535
K257	HA	1H	4.294
K257	HB2	1H	1.806
K257	HB3	1H	1.721
K257	HD2	1H	1.634
K257	HE2	1H	2.947
K257	HG2	1H	1.432
K257	HG3	1H	1.385
S258	H	1H	8.410
S258	HA	1H	4.347
S258	HB2	1H	3.829
S258	HB3	1H	3.797
K259	H	1H	8.551
K259	HA	1H	4.310
K259	HB2	1H	1.820
K259	HB3	1H	1.708
K259	HD2	1H	1.634
K259	HE2	1H	2.946
K259	HG2	1H	1.412
K259	HG3	1H	1.369
I260	H	1H	8.302
I260	HA	1H	4.092
I260	HB	1H	1.810
I260	HD1	1H	0.822
I260	HG12	1H	1.462
I260	HG13	1H	1.167
I260	HG2	1H	0.88
G261	H	1H	8.666
G261	HA3	1H	3.959
S262	H	1H	8.328
S262	HA	1H	4.497
S262	HB2	1H	3.916
S262	HB3	1H	3.836
T263	H	1H	8.453
T263	HA	1H	4.319
T263	HB	1H	4.277
T263	HG2	1H	1.175
E264	H	1H	8.465
E264	HA	1H	4.191
E264	HB2	1H	1.979
E264	HB3	1H	1.890
E264	HG2	1H	2.226
E264	HG3	1H	2.201
N265	H	1H	8.533
N265	HA	1H	4.613
N265	HB2	1H	2.793
N265	HB3	1H	2.692
N265	HD21	1H	7.682
N265	HD22	1H	6.964
L266	H	1H	8.236
L266	HA	1H	4.242
L266	HB2	1H	1.591
L266	HB3	1H	1.481
L266	HD1	1H	0.878
L266	HD2	1H	0.807
L266	HG	1H	1.350
K267	H	1H	8.275
K267	HA	1H	4.188
K267	HB2	1H	1.714
K267	HB3	1H	1.683
K267	HD2	1H	1.599
K267	HE2	1H	2.921
K267	HG2	1H	1.345
K267	HG3	1H	1.268
H268	H	1H	8.347
H268	HA	1H	4.581
H268	HB2	1H	3.157
H268	HB3	1H	3.056
H268	HD2	1H	7.086
H268	HE1	1H	7.623
